# Unique Phenotypes With Corresponding Pathology in Late-Onset Hereditary Transthyretin Amyloidosis of A97S vs. V30M

**DOI:** 10.3389/fnagi.2021.786322

**Published:** 2022-01-26

**Authors:** Hsueh-Wen Hsueh, Chi-Chao Chao, Koping Chang, Yung-Ming Jeng, Masahisa Katsuno, Haruki Koike, Sung-Tsang Hsieh

**Affiliations:** ^1^Department of Neurology, National Taiwan University Hospital, Taipei, Taiwan; ^2^Department of Anatomy and Cell Biology, National Taiwan University College of Medicine, Taipei, Taiwan; ^3^Department of Pathology, National Taiwan University Hospital, Taipei, Taiwan; ^4^Department of Neurology, Nagoya University Graduate School of Medicine, Nagoya, Japan; ^5^Graduate Institute of Brain and Mind Sciences, National Taiwan University College of Medicine, Taipei, Taiwan; ^6^Center of Precision Medicine, National Taiwan University College of Medicine, Taipei, Taiwan

**Keywords:** hereditary transthyretin amyloidosis (ATTRv), carpal tunnel syndrome, dysphagia, amyloid deposition, natural course

## Abstract

**Objective:**

Hereditary transthyretin amyloidosis (ATTRv) encompasses different phenotypes among various genotypes. The analysis of the natural history and risk factors of faster progression in different genotypes would refine the treatment strategy.

**Methods:**

The clinical manifestations of ATTRv from A97S (p.A117S) of Taiwanese and late-onset V30M (p.V50M) of Japanese were compared. An autopsy study of A97S was performed.

**Results:**

There existed three unique features in the A97S cohort compared to the V30M cohort: (1) dysphagia, (2) carpal tunnel syndrome (CTS), and (3) onset age. First, dysphagia was common in A97S (53.4%) but not in V30M and served as a contributor to fast disease progression. All phases of swallowing were affected. In the autopsy pathology, there were extensive amyloid deposits in the viscera and nerves of the tongue, larynx, and esophagus. In A97S, 45 patients (43.3%) had a history of CTS before the onset of length-dependent symptoms by 3 years. The amyloid deposition was more prominent in the median nerve than that in the transverse carpal ligament. The onset age at different stages was younger in the A97S cohort than the V30M cohort by 4–5 years.

**Conclusion:**

These phenotypic characteristics together with autopsy pathology in A97S are distinct from V30M. Early dysphagia in A97S correlated with fast progression. In A97S, median neuropathy leading to CTS might be in a continuous spectrum of ATTRv course rather than an independent disease entity. Such observations may serve as a foundation to explore and analyze unique phenotypes among various genotypes.

## Introduction

Various mutations of hereditary transthyretin amyloidosis (ATTRv) or familial amyloid polyneuropathy (FAP), which affects motor, sensory, and autonomic nerves, encompass different phenotypes (Ando et al., 2013; Carr et al., 2016; Sridharan et al., 2018; Adams et al., 2019; Damy et al., 2019; Dang et al., 2019; Lane et al., 2019). While the V30M (p.V50M) (Benson et al., 2020) with polyneuropathy is the most common pathogenic variant in Japan and around the world (Ikeda et al., 2002; Adams et al., 2015; Coelho et al., 2018; Yamashita et al., 2019), A97S (p.A117S) (Benson et al., 2020) constitutes the most frequent mutation in Taiwan and has been reported in China (Du et al., 2021), Malaysia (Low et al., 2019), and Thailand (Pasutharnchat et al., 2021). V30M in Japan had two onset patterns, namely, early- vs. late-onset type (Koike et al., 2002, 2004, 2012), and the latter was also reported in Portugal and France (Conceição and De Carvalho, 2007; Dohrn et al., 2013; Buades-Reinés et al., 2016; Pinto et al., 2019). In contrast, the onset age of A97S in Taiwan was generally after the 5th decade (Chao et al., 2019). Under the umbrella of the late-onset ATTRv, it is unknown which features were different between A97S and V30M. Furthermore, given the development of new disease-modifying therapies, it is crucial to identify risk factors for symptomatic ATTRv at the early stage of the disease to initiate treatments (Castaño et al., 2012; Berk et al., 2013; Adams et al., 2018, 2019; Benson et al., 2018; Maurer et al., 2018; Groothof et al., 2020). Thus, it is important to analyze the natural history of the disease and, in particular, risk factors for early-onset and accelerating progression by the same measurements among different genotypes.

In ATTRv, there are certain manifestations, serving as prodrome symptoms, but there were not examined extensively and even correlated with autopsy pathology, such as dysphagia and carpal tunnel syndrome (CTS). Dysphagia is an intriguing symptom of ATTRv, and amyloid deposits in lower cranial nerves were reported as the possible cause (Ikeda et al., 1998; Obici and Suhr, 2019). However, the frequency of dysphagia in different genotypes remains elusive. An integrated study incorporating clinical profiles, functional study of swallowing, and pathology is needed to clarify (1) the underlying pathophysiology and (2) its effect on the natural course of ATTRv.

Traditionally, CTS in ATTRv is considered as an entrapment neuropathy of the median nerve by the compression of the surrounding tissue, and amyloid was detected in the transverse carpal ligament (Samões et al., 2017). However, the amyloid deposition of the wild-type TTR was also reported in the transverse carpal ligament of patients with idiopathic CTS (Gioeva et al., 2013). Furthermore, the response to carpal tunnel release surgery in CTS of ATTRv was impersistent (Ando et al., 2013). All these observations prompted us to examine: (1) the mechanisms of CTS in ATTRv and (2) the clinical significance of CTS in ATTRv, i.e., whether it reflects a state or disease progression.

To address the above issues, this study analyzed the clinical profiles and laboratory tests of the two cohorts: A97S of Taiwanese and the late-onset V30M of Japanese to investigate the similarities and differences between these two cohorts. Furthermore, an autopsy case of A97S provided pathology underlying the mechanisms of the unique symptomatology of dysphagia and CTS.

## Materials and Methods

### Patient Recruitment

We recruited patients with ATTRv referred to the Department of Neurology, National Taiwan University Hospital, Taipei, Taiwan, and the Department of Neurology, Nagoya University Graduate School of Medicine, Nagoya, Japan. The evaluation of V30M cases was performed before 2012, and the Taiwanese cases were recruited until July 2020. Part of the clinical features and their course in V30M patients has been reported elsewhere (Koike et al., 2012). All patients were confirmed with the genetic diagnosis of A97S (p.A117S according to the 2020 updated nomenclature recommendation) (Benson et al., 2020) in Taiwanese and V30M (p.V50M) in Japanese with an age of onset older than 50 years (late-onset V30M, abbreviated as V30M in the following description). This retrospective study was approved by the Ethics Committee of the National Taiwan University Hospital, Taipei, Taiwan (ID: 201404014RINB, 201908065RINA), and the Nagoya University Graduate School of Medicine, Nagoya, Japan (ID: 2014-0424). Informed consent was obtained from all patients participating in the study.

### Clinical Evaluations

All patients had detailed evaluations such as history taking, neurological examinations, and laboratory tests. The laboratory tests included a nerve conduction study and a blood test. In addition, 84 A97S patients and 39 V30M patients had accepted the cerebrospinal fluid (CSF) analysis. The neuropathy manifestations included motor, sensory, and autonomic symptoms, but the symptoms related to common entrapment neuropathy other than CTS or radiculopathy were excluded. The upper limb onset was defined as the motor and sensory symptoms other than CTS occurring at the upper limbs, and the asymmetric onset was defined as the interval of neuropathy symptoms that happened from one side to bilateral sides was longer than 3 months. Relevant information included: (1) paternal or maternal inheritance, (2) negative (numbness) and positive (tingling, spontaneous pain, and allodynia) sensory symptoms, and (3) autonomic symptoms (gastrointestinal symptoms: constipation, diarrhea, or alternating constipation and diarrhea, urinary symptoms, orthostatic dizziness, and erectile dysfunction).

Functional disability was evaluated according to a FAP stage score of 0–4 modified from the Coutinho’s staging system (Adams et al., 2016): 0, normal; 1, having mild neuropathy symptoms such as motor, sensory, or autonomic, but with an independent ambulance; 2, having major motor disturbances and needing support to walk; 3, being confined to a wheelchair or bed; and 4, death. We collected the onset time of CTS and each FAP stage. However, some patients in the A97S cohort (39/105, 37.1%) would use diflunisal (Castaño et al., 2012; Berk et al., 2013), tafamidis (Maurer et al., 2018), or small interference RNA (Adams et al., 2018) after the medication was available or lose follow-up, so we right-censored the data at the time of (1) starting medication or (2) losing follow-up for more accurate analysis of the natural course.

### Cardiac Assessment

Because the early cardiac symptoms may be subtle or non-specific, we collected the first objective evidence rather than self-reported first symptoms. The first objective evidence means (1) patient have no symptoms but was examined after ATTRv was diagnosed or (2) patients have the symptoms that the clinician considered to be related to the heart. We evaluated the heart involvement by cardiac sonography, ECG, and 24-h Holter monitor. The cardiac sonography measured the thickness of the interventricular septum and posterior wall. The thickness greater than or equal to 1.3 cm was defined as ventricular hypertrophy. The sparkling myocardium on cardiac sonography was diagnosed as scattered distinct hyperechoic signals in the myocardium. In the heart rhythm assessed by Holter monitor and ECG, the abnormality was considered if (1) more than 100 supraventricular or ventricular ectopy were recorded in 1 day, (2) long pause over 3 s, and (3) arrhythmia such as atrial fibrillation, atrioventricular conduction block, intraventricular conduction block, or pacemaker rhythm.

### Swallowing Studies in Patients With A97S Cohort

The swallowing function was evaluated by several functional studies such as videofluoroscopic swallowing study, esophageal transit scintigraphy, and sialoscintigraphy. In the videofluoroscopic swallowing study, patients were asked to swallow different quantities of barium suspension (thin: water; thick: honey-thick; and paste: pudding thick). The fluoroscopy would record the time of oral transit, pharyngeal transit, and swallowing triggers. The presence of penetration or aspiration was also recorded. Abnormal hyoid bone elevation was defined as the elevation that was lower than the mandible angle. In the esophageal transit scintigraphy, patients were asked to swallow radiotracer (1 millicurie technetium-99m phytate in 15 ml of water) in upright and supine positions, respectively. The esophageal transit time longer than 15 s was considered abnormal. In the sialoscintigraphy, patients accepted intravenous injection of 10 millicurie technetium-99m sodium pertechnetate, and serial anterior images were acquired every 60 s for 30 min. The accumulation function of salivary glands was evaluated in the first 15 min. The patient accepted oral ascorbic acid 500 mg at the time of 15 min to evaluate the excretory function of salivary glands. The accumulation and discharge curves were recorded.

### Pathology Studies From an Autopsy Patient With A97S

We performed an autopsy of a patient with ATTRv. All procedures were approved by the National Taiwan University Hospital Research Ethics Committee, and informed consent was obtained from the patient and next of kin for body donation. Fixed organ and nerve tissues were harvested. The histological examinations included: (1) routine H&E staining on 4-μm paraffin-embedded sections and (2) Congo red staining on 10-μm paraffin-embedded sections.

### Electron Microscopy of Sural Nerve Biopsies

The assessment of nerve pathology followed our established protocol (Chiang et al., 2005). The sural nerves were collected at the level of the trifurcation of the sciatic nerve and then fixed in 5% glutaraldehyde in 0.1 M phosphate buffer at 4°C overnight. The tissues were post-fixed in 2% osmic acid for 2 h at room temperature, dehydrated, and embedded in Epon 812 resin (Polysciences, Philadelphia, PA, United States). Ultrathin (70 nm) sections were cut on a Reichert Ultracut E (Leica, Wetzlar, Germany), stained with uranyl acetate and lead citrate, and observed under a transmission electron microscope (H7100, Hitachi, Tokyo, Japan).

### Statistical Analysis

Continuous variables, expressed as means ± SDs, were compared using the *t*-test if they followed a normal distribution (by Shapiro–Wilk test). The non-parametric test was used if the variables did not follow the normal distribution. The chi-square test or Fisher’s exact test were used to compare the categorical data. We used univariate linear regression and ANOVA to examine the contribution of the clinical factors to the onset of FAP stage 1. The Kaplan–Meier survival analysis was used to present the survival curves. The Cox proportional hazard model was used to evaluate the association of progression rate with baseline characteristics and clinical variables with “entering the next stage” as an event. Outliers were considered when the value is larger than the third quantile plus 1.5*interquartile range (IQR) or smaller than the first quantile minus 1.5*IQR. All analyses were performed using Stata software (StataCorp LP, College Station, TX, United States). The results were considered significant at a value of *P* < 0.05.

## Results

### Unique Manifestations: A97S vs. V30M

This study recruited subjects with confirmed TTR genotypes: A97S (105 Taiwanese patients) and late-onset V30M (50 Japanese patients) with the age of onset (entering FAP stage 1) ≥ 50 years. The clinical phenotypes and laboratory findings are summarized in Tables 1, 2 and Supplementary Table 1. There were two unique features in the A97S cohort, namely, dysphagia and CTS.

**TABLE 1:** Clinical profiles in hereditary transthyretin amyloidosis.

	A97S of Taiwan (*N* = 105) *n* (%)	V30M of Japan (*N* = 50) *n* (%)	*P*
Male	78/105 (74.3%)	43/50 (86%)	0.099
Presence of dysphagia	55/103 (53.4%)	1/50 (2%)	<0.001*
Upper limb onset excluding CTS	27/105 (25.7%)	5/50 (10.0%)	0.024*
**Initial symptom of amyloidosis**			
Autonomic	17/105 (16.2%)	5/50 (10.0%)	0.302
Sensory	65/105 (61.9%)	33/50 (66.0%)	0.621
Motor	7/105 (6.7%)	3/50 (6.0%)	0.875
Motor and sensory	13/105 (12.4%)	4 (8%)	0.415
Heart	3/105 (2.9%)	2/50 (4.0%)	0.658
Ocular	0/105 (0%)	3/50 (6.0%)	0.032*
**Onset age of the symptoms**			
motor symptom	62.2 ± 6 (*n* = 105)	66.3 ± 6.2 (*n* = 42)	0.0006*
sensory symptom	61.2 ± 6.3 (*n* = 102)	65.0 ± 6.5 (*n* = 50)	0.0008*
autonomic symptom	62.3 ± 6.8 (*n* = 103)	66.4 ± 6.3 (*n* = 43)	0.0021*
heart with laboratory evidence	64.2 ± 6.3 (*n* = 89)	69.3 ± 6.2 (*n* = 23)	0.0006*
**Sensory symptom**			
Numbness	105/105 (100%)	50/50 (100%)	-
Spontaneous pain	75/103 (73.5%)	34/50 (68.0%)	0.477
**Autonomic symptoms**			
Gastrointestinal symptom	89/105 (84.8%)	40/50 (80.0%)	0.458
Urinary symptoms	45/105 (42.9%)	24/50 (48.0%)	0.547
Orthostatic dizziness	69/105 (66.3%)	27/50 (54.0%)	0.139
Erectile dysfunction	40/78 (51.3%)	10/21 (47.6%)	0.846-
**Death etiology**			
Heart failure	23/47 (48.9%)	8/21 (38.0%)	0.407
Cachexia with secondary infection	9/47 (19.1%)	4/21 (19.0%)	1.000
Sudden death	7/47 (14.9%)	7/21 (33.3%)	0.082

*CTS, Carpal tunnel syndrome.*

*The history of CTS was defined according to the following two criteria: (1) The patient had been diagnosed and treated by a specialist and (2) The symptom improved after treatment such as rehabilitation and surgery.*

*P < 0.05.

**TABLE 2:** Laboratory findings at diagnosis.

	A97S of Taiwan (*N* = 105) *n* (%)	V30M of Japan (*N* = 50) *n* (%)	*P*
**Sonography of heart evaluation**			
Ventricular hypertrophy	74/94 (82.2%)	18/27 (66.7%)	0.196
Sparkling pattern	37/92 (42%)	12/24 (50%)	0.388
**Cardiac rhythm**			–
Frequent supraventricular or ventricular ectopy^a^	41/80 (51.3%)	0/5 (0%)	0.056
Long pause > 3 s	1/80 (1.3%)	0/5 (0%)	1.000
Arrhythmia*	42/80 (52.5%)	5/6 (83.3%)	0.215
Any abnormality	65/80 (81.3%)	5/8 (62.5%)	0.155
Pacemaker implantation	11/105 (10.5%)	7/50 (14%)	0.522
Cerebrospinal fluid protein (mg/dL)	64.9 ± 28.8	56.2 ± 29.8	0.024
Serum Creatine > 1.5 mg/dL	2/98 (2%)	0/36 (0%)	1.000
**Nerve conduction studies**			
**Median nerve**			
Mean MCV (m/s)	43.4 ± 6.8	45.2 ± 9.8	0.164
Mean DL (m/s)	6.2 ± 1.3	5.3 ± 1.7	<0.001^#^
Mean CMAP (m/s)	2.5 ± 2	3.3 ± 2.8	0.032^#^
Mean SCV (m/s)	40.8 ± 8.7	46.6 ± 5.8	0.863
Mean SNAP (uV)	2.8 ± 4.7	2.3 ± 3.6	0.006^#^
Mean Terminal latency index	0.32 ± 0.06	0.37 ± 0.1	<0.001^#^
Terminal latency index < 0.35	53/73 (72.6%)	17/45 (37.8%)	<0.001^#^
**Tibial nerve**			
MCV (m/s)	36.3 ± 6.2	37.1 ± 5.6	0.506
DL (m/s)	5 ± 1.9	5.8 ± 1.4	<0.001^#^
CMAP (m/s)	1.5 ± 2.4	0.5 ± 0.9	<0.001^#^
**Sural nerve**			
SCV (m/s)	44 ± 6.1	38.1 ± 5.2	0.595
SNAP (m/s)	1.1 ± 3.2	0.6 ± 1.6	0.012^#^

*^a^Over 100 supraventricular or ventricular ectopy in one day.*

* Arrhythmia such as atrial fibrillation, atrioventricular conduction block, or intraventricular conduction block. MCV, motor conduction velocity; DL, distal latency; CMAP, compound motor action potential; SCV, sensory conduction velocity; SNAP, sensory nerve action potential.

*^#^ P < 0.05.*

### Dysphagia in A97S

Dysphagia was a distinct symptom in A97S compared to V30M (53.4 vs. 2.0%, *P* < 0.001). Associated symptoms in A97S included choking (43.6%) and hoarseness (31.7%). Aspiration pneumonia was noted in 19.4% of the A97S patients. Among the 56 A97S patients entering FAP stage 1, 22 patients had dysphagia, choking, or hoarseness before the diagnosis. Fourteen patients had completed examinations for dysphagia (Table 3). There was no structural lesion grossly in these patients. The videofluoroscopic swallowing study revealed oropharyngeal dysphagia in these patients. The transit times of the oral phase and pharyngeal phase were all prolonged when the patients swallowed liquids with various contents, but the swallowing trigger time was not prolonged. The penetration or aspiration was also noted, and the level of hyoid bone elevation during swallowing was lower than that of the mandible angle. The transit time of the esophageal phase was prolonged in all patients in the supine position but was prolonged in only one patient in the upright position. Notably, the non-peristaltic contraction was noted in 2 patients. The sialoscintigraphy revealed accumulation or excretion dysfunction in salivary glands. These functional studies were complementary to dysphagia symptoms that developed at the early stage of A97S. We also compared the laboratory findings between the patients of ATTRA97S entering FAP stage 1 with dysphagia and those without dysphagia (Supplementary Table 2). Notably, the mean of terminal latency index, abnormal terminal latency index rate, and the sural sensory nerve action potential were worse in the patients without dysphagia.

**TABLE 3:** Examinations of dysphagia in the A97S cohort.

Examinations	Median (range)	Abnormal rate	Norm
**Video fluoroscopic swallowing study (*n* = 8)**			
Oral transit time (thin)	2.91 (0.5–45.38)	4 (50%)	<2 s
Oral transit time (thick)	1.85 (0.44–10.41)	4 (50%)	<2 s
Oral transit time (paste)	3.65 (0.63–27.87)	6 (75%)	<2 s
Pharyngeal transit time (thin)	1.62 (1.16–2.62)	8 (100%)	<1 s
Pharyngeal transit time (thick)	1.29 (0.9–3.13)	7 (87.5%)	<1 s
Pharyngeal transit time (paste)	1.33 (1.15–5.97)	7 (100%)	<1 s
Swallowing triggers (thin)	0.285 (0.17–1.85)	2 (25%)	<1 s
Swallowing triggers (thick)	0.18 (0.13–0.54)	0 (0%)	<1 s
Swallowing triggers (paste)	0.26 (0.1–4.71)	1 (12.5%)	<1 s
Penetration or aspiration		3 (37.5%)	
Abnormal hyoid bone elevation*		2 (25%)	
**Esophageal transit time (ETT) (*n* = 4)**			
Prolonged ETT in upright position		1 (25%)	<15 s
Prolonged ETT in supine position		4 (100%)	<15 s
Non-peristaltic contractions		2 (50%)	
**Sialoscintigraphy (*n* = 5)**			
Excretory dysfunction		2 (40%)	
Accumulation dysfunction		3 (60%)	

*Abnormal hyoid bone elevation is defined as the elevation that was lower than the mandible angle.

**FIGURE 1:**
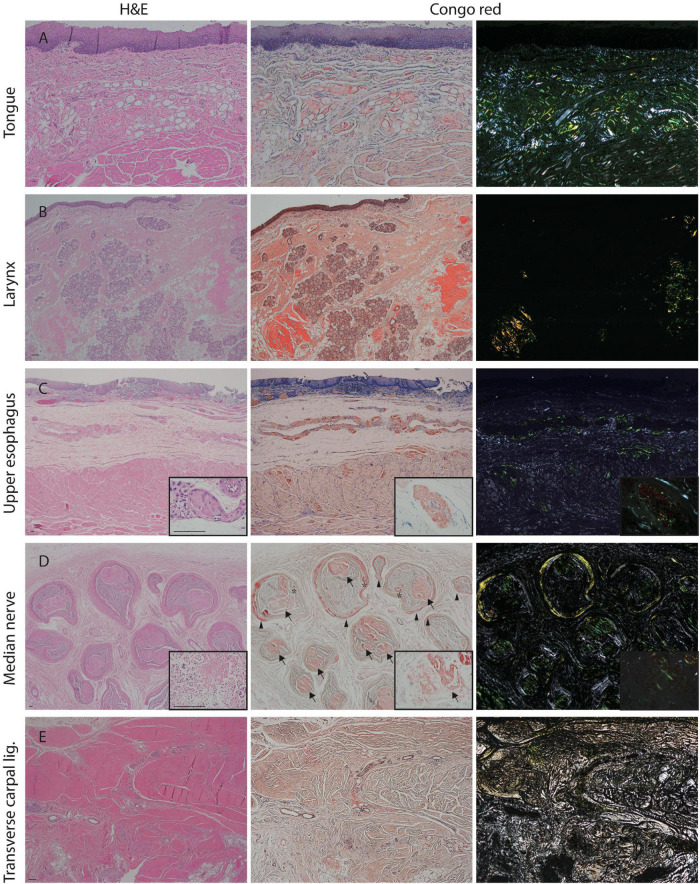
Amyloid pathology of the median nerve and visceral organs from an autopsy of A97S patient. Amyloid deposits were demonstrated in the tongue **(A)**, laryngeal wall **(B)**, and upper esophageal wall **(C)** including in the submucosal nerve fibers (**C**, inset). Amyloid deposits were also abundant in the endoneurium and perineurium of the median nerve at the level of carpal tunnel (**D**, inset). In contrast, the amyloid deposition was much less in the transverse carpal ligament **(E)**. Arrow: amyloid deposits in the endoneurium; Arrowhead: amyloid deposits in the perineurium; asterisk: amyloid deposits in the vascular wall. First column: H&E staining; Second column: Congo red staining under a regular light microscope; Third column: Congo red staining under a polarized microscope. Scale bars = 50 μm.

We further investigated the underlying pathology in an autopsy case of A97S. There were marked amyloid deposits in the tongue, larynx, and esophagus and submucosal nerve fibers (Figures 1A–C), providing microscopical evidence as to the mechanisms of amyloid pathology in the swallowing structures and innervation during the oral phase, pharyngeal phase, and esophageal phase.

### Carpal Tunnel Syndrome in A97S

In the A97S cohort, CTS was a unique feature compared to the V30M cohort: 43.3% had CTS confirmed by a neurologist according to nerve conduction studies or a history of carpal tunnel release surgery. The onset age of symptomatic CTS was younger than the length-dependent symptoms (57.6 ± 6.3 vs. 60.6 ± 6.3, *P* = 0.004) in the A97S cohort. Despite the operations, most symptoms were only mildly and transiently alleviated. The diagnosis of CTS was further validated by neurophysiological evidence of reduced terminal latency index in A97S compared to that in V30M (0.32 ± 0.06 vs. 0.37 ± 0.10, *P* < 0.001). A terminal latency index smaller than 0.35 served as a criterion to diagnose CTS (Dumitru et al., 2002). The phenomenon of more frequent CTS in A97S was further substantiated by a higher proportion of A97S with abnormal terminal latency index than V30M (72.6 vs. 37.8%, *P* < 0.001, Table 2). Such an observation corroborated with our report of the median nerve neuropathy as an early manifestation of A97S (Chiang et al., 2021). Furthermore, we had the opportunity to examine an autopsy of an A97S patient. The pathology showed much amyloid deposition in the endoneurial area of the median nerve at the level of carpal tunnel in comparison with the relatively scant amount of amyloids in the transverse carpal ligament (Figures 1D,E), providing evidence of amyloid pathology in the median nerve and contributing to the development of CTS.

### Phenotypic Characterization of A97S vs. V30M: Distinct and Shared Features in Amyloid Fibril Ultrastructure and Progression

**FIGURE 2:**
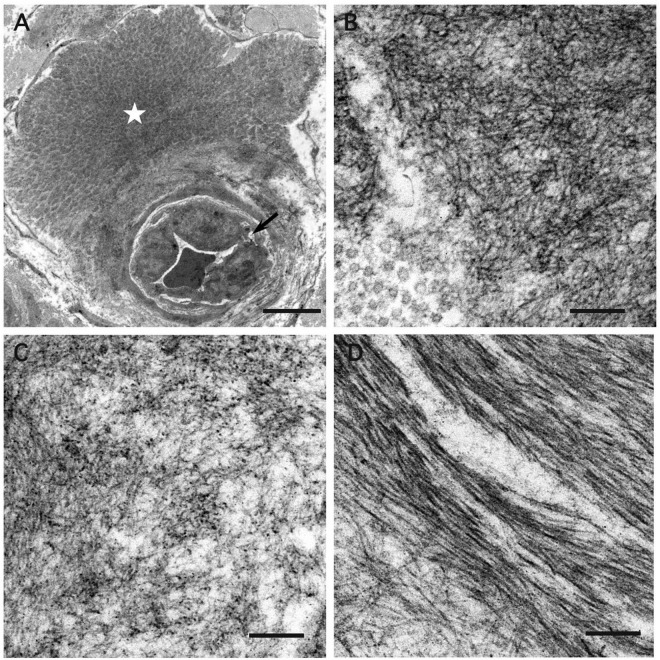
Electron microscopic findings of amyloid fibrils in the endoneurium. Cross-sections of sural nerve biopsy specimens from patients with hereditary transthyretin amyloidosis (ATTRv). **(A,B)** A97S patients from Taiwan, **(C)** Late-onset V30M patient from a non-endemic area of Japan, and **(D)** Early-onset V30M patient from an endemic focus of Japan. A microvessel indicated by an arrow is apposed by a mass of amyloid fibrils (asterisk) **(A)**. Amyloid fibrils are generally short and thin in the A97S and late-onset V30M patients **(B,C)**, while they tend to be long in the early-onset V30M patient **(D)**. Uranyl acetate and lead citrate stain. Scale bars = 5 μm **(A)** and 0.2 μm **(B–D)**.

The different genotypes of A97S vs. V30M yielded distinct amyloid patterns in nerve biopsies observed under electron microscopy (Figure 2). Amyloid fibrils were generally short and thin in the A97S (*n* = 20) and late-onset V30M patients (*n* = 32). These patterns were in contrast to that of the early-onset V30M (*n* = 6), which tended to be long.

The distribution of patients at different stages was similar (*P* = 0.13) at the time of the first referral to our medical centers. In the A97S cohort, there were 49 patients entering FAP stage 3, 22 patients stayed in FAP stage 2, and 34 patients stayed in FAP stage 1. In the V30M group, 31 patients entered FAP stage 3, 5 patients stayed in FAP stage 2, and 14 patients stayed in FAP stage 1. The backgrounds and clinical characteristics are listed in Tables 1, 2. Among the patients with sudden death in both cohorts, only one patient in the A97S cohort had pacemaker implantation. In summary, there were more patients with neuropathic symptoms starting from upper limbs (excluding CTS) in A97S patients than late-onset V30M patients (25.7 vs. 10.0%, *P* = 0.024), and other clinical manifestations were similar between these two cohorts. Three patients in the V30M cohort had ocular symptoms as the onset symptom, but this was not observed in the A97S cohort. All three Japanese patients with ocular symptoms accepted operation for vitreous opacities, and amyloid deposits were detected in one patient.

### Natural Course of Hereditary Transthyretin Amyloidosis Between A97S and V30M: Onset and Progression

**FIGURE 3:**
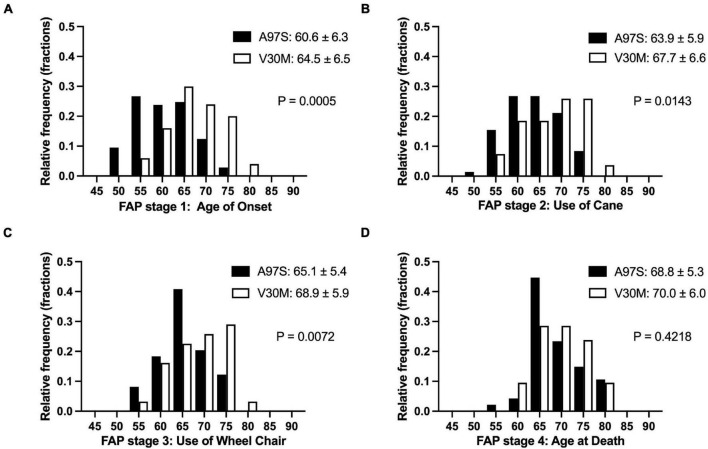
The natural course of ATTRv of A97S and V30M: comparing the histogram of the age in each familial amyloid polyneuropathy (FAP) stage. The histograms of the age for each FAP stage are depicted between A97S Taiwanese vs. V30M Japanese patients **(A–D)**. At all stages, the A97S cohort had earlier onset age, i.e., entering FAP stage 1 **(A)**, and the age entering successive FAP stage **(B–D)**.

**FIGURE 4:**
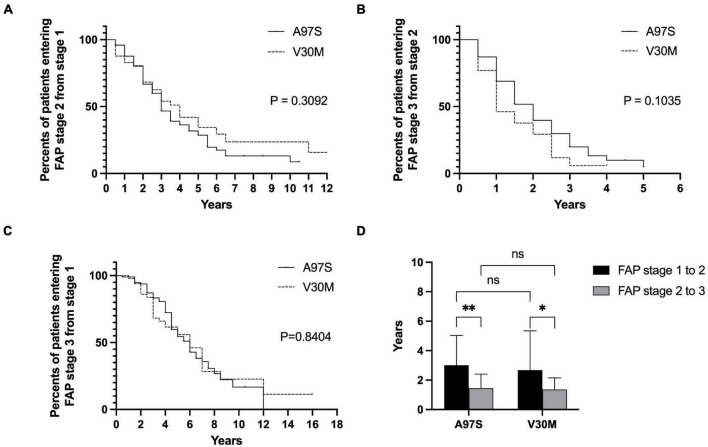
The disease progression in ATTRv between A97S and V30M according to the interval of FAP stage from 1 to 2, 2 to 3, and 1 to 3. There was no difference in the Kaplan–Meier survival curve between A97S and V30M according to the analysis of the interval entering stage 2 from stage 1 **(A)**, in the interval entering stage 3 from stage 2 **(B)**, and in the interval entering stage 3 from stage 1 **(C)**. **(D)** The interval of FAP stage 1–2 was longer than the interval of FAP stage 2–3 in both A97S and V30M ATTRv cohorts (A97S: 3.0 ± 2.0 years vs. 1.4 ± 1.0 years, *P* < 0.0001; V30M: 2.7 ± 2.7 years vs. 1.4 ± 0.8 years, *P* = 0.04). *P < 0.05; **P < 0.01; ns, no significant differences.

The onset age was different between A97S and V30M, i.e., the age of onset and the age of entering FAP stage 2 and FAP stage 3 were younger in A97S patients than in V30M patients (Figure 3), but the interval from FAP stage 1 to 2, 2 to 3, and 1 to 3 was similar between these two cohorts (Figures 4A–C). We further analyzed the interval of FAP 1–2 and 2–3 in patients who had entered into FAP stage 3. The disability became accelerating when entering into FAP stage 2, i.e., the interval of FAP 2–3 was shorter than that of FAP 1–2 in both cohorts (A97S: 3.0 ± 2.0 years vs. 1.4 ± 1.0 years, *P* < 0.0001; V30M: 2.7 ± 2.7 years vs. 1.4 ± 0.8 years, *P* = 0.04) (Figure 4D).

### Factors Affecting the Onset Age of Hereditary Transthyretin Amyloidosis

We then explored the factors affecting the onset age of ATTRv with linear regression and ANOVA. The univariate analysis showed that the male sex was related to the earlier onset in A97S patients but such a difference did not exist in the late-onset V30M patients [β coefficient (95% CI): A97S: -4.8 (-7.5 to -2.2) vs. V30M: -0.37 (-5.78 to 5.04)]. In contrast, there was no effect on the onset age from whether the upper limb onset neuropathic symptoms [β coefficient (95% CI): A97S: -2.3 (-5 to 0.47) vs. V30M: 0.9 (-4.87 to 6.67)] or paternal inheritance [β coefficient (95% CI): A97S: 2.3 (-1.7 to 6.3) vs. V30M: -5.43 (-14.3 to 3.49)]. The ANOVA analysis revealed no significant effect on the onset age in the respect of initial symptoms of ATTRv in both cohorts.

### Factors Affecting the Progression of Hereditary Transthyretin Amyloidosis

To understand the clinical factors contributing to disease progression, we analyzed patients who had been evaluated at FAP stage 1 and 2 with the Cox hazard model after excluding the outliers (51 A97S patients and 26 V30M patients who had been evaluated at FAP stage 1, and 34 A97S patients and 8 V30M patients who had been evaluated at stage 2). These factors affecting the progression rate of ATTRv are summarized in Table 4. In the univariate analysis of A97S patients, the early presence of dysphagia was related to a faster progression rate from stage 1 to 2. However, these risk factors in the A97S cohort were not noted in the V30M cohort.

**TABLE 4:** Clinical factors affecting the progression in ATTRv.

	ATTRA97S in Taiwan		ATTRV30M in Japan	
Univariate Cox hazard model	Stage 1 → 2 (*n* = 51)	Stage 2 → 3 (*n* = 34)	Stage 1 → 2 (*n* = 23)	Stage 2 → 3 (*n* = 8)
Male sex	5.93 (0.78–44.9)	0.99 (0.21–4.74)	1.35 (0.17–10.8)	1.57 (0.18–14.1)
ATTRv onset age	0.99 (0.91–1.07)	0.95 (0.86–1.05)	0.97 (0.87–1.09)	1.05 (0.96–1.15)
Upper limb onset	0.67 (0.26–1.75)	1.43 (0.44–4.58)	1.11 (0.14–8.93)	1.33 (0.12–14.7)
Paternal inheritance	0.40 (0.10–1.56)	0.18 (0.02–2.05)	-^#^	-^#^
Weight loss	1.48 (0.61–3.58)	1.00 (0.34–23.01)	1.16 (0.34–4.04)	1.09 (0.10–12.1)
Body mass index	0.90 (0.79–1.03)	0.84 (0.63–1.12)	0.84 (0.35–2.02)	1.00 (0.45–2.21)
Dysphagia	3.89 (1.61–9.40)*	1.02 (0.32–3.26)	-^#^	-^#^
CSF protein (mg/dL)	0.99 (0.97–1.01)	1.01 (0.99–1.02)	0.99 (0.97–1.02)	1.00 (0.96–1.03)
CSF protein (>60 mg/dL)	0.80 (0.33–1.95)	1.46 (0.47–4.49)	1.40 (0.25–7.82)	1.15 (0.21–6.29)

*Data expressed as hazard ratio (95% CI).*

*CSF, cerebrospinal fluid; HR, hazard ratio.*

*P < 0.05.

*^#^No patients had the symptoms, or the patient number was insufficient to calculate the hazard ratio.*

## Discussion

This study demonstrated the unique clinical phenotypes between two genotypes of the A97S and the late-onset V30M, i.e., more frequent dysphagia, CTS, and earlier onset age in A97S. Furthermore, the distinct symptoms of dysphagia and CTS were evidenced by amyloid deposition in the visceral organs and nerves in the autopsy pathology. Such a combination of phenotype analysis and pathology examinations may serve as a foundation for future analysis in other genotypes. In addition to being a late-onset ATTRv, both cohorts shared similarities in (1) the majority of clinical symptoms and (2) the ultrastructure of the amyloid fibril, i.e., the length was comparable in A97S and late-onset V30M but shorter than the early-onset V30M Japanese cohort.

The A97S cohort had a unique symptom of dysphagia. Despite the report of this symptom (Ikeda et al., 1998; Obici and Suhr, 2019), dysphagia was not yet systemically examined in other genotypes (Monteiro et al., 2012). About half of the A97S patients had dysphagia, and dysphagia symptoms could be present at the early stage of ATTRv, which was rarely reported in other etiology of polyneuropathy. Furthermore, the presence of dysphagia at an early stage would suggest a faster progression in the A97S cohort. Swallowing could be divided into the oral phase, pharyngeal phase, and esophageal phase, and the videofluoroscopic swallowing study performed in part of ATTRA97S patients with dysphagia indicated that all these three phases were affected. Most patients mainly presented with dysphagia at the pharyngeal phase with choking. Previously, the cause of dysphagia was attributed to amyloid deposits in the hypoglossal nerve or vagus nerve (Ikeda et al., 1998; Obici and Suhr, 2019). Importantly, the A97S autopsy revealed extensive amyloid pathology in the tongue and larynx, providing pathological evidence that the structures governing swallowing were affected.

This study provided unique features and pathological data for CTS in A97S compared with the variable frequencies of CTS in different genotypes (Mariani et al., 2015; Milandri et al., 2020). Some patients had carpal tunnel release surgery, but the effect was minimal. This study provided the pathological evidence of amyloid deposits in the median nerve from an A97S autopsy, which likely contributed to the pathophysiology of CTS in A97S. Although amyloid that was deposited in the transverse carpal ligament was proposed as the cause of CTS in ATTRv (Samões et al., 2017), the minimal effect of carpal tunnel release surgery raised an alternative hypothesis. In A97S, the more prominent amyloid deposition in the median nerve than that in the transverse carpal ligament implied a direct effect of amyloid deposition on the median nerve serving as the pathogenesis of CTS. This amyloid pathology in the median nerve was different from the CTS in the general population or the compression hypothesis (Samões et al., 2017). Accordingly, the amyloid pathology in the median nerve at the wrist potentially prompted the median nerve vulnerable to the compression from surrounding tissue of the transverse carpal ligament (Koike et al., 2011). Such an observation also explains the observation of minimal or transient effect after release surgery (Ando et al., 2013). Furthermore, this study documented that the CTS onset was earlier than the onset of the length-dependent neuropathy symptoms. The observation may provide the explanation of our recent observation, i.e., the median neuropathy in the carriers of ATTRv was correlated with lower intraepidermal nerve fiber density and more abnormal electrophysiological parameters, and the median conduction parameters could serve as a surrogate marker of neuropathy involving both large- and small-fiber nerves in A97S carriers (Chiang et al., 2021). In summary, CTS in the A97S cohort may be in a continuous spectrum of ATTRv course rather than an independent disease entity.

Several unique features distinguished these two cohorts (A97S vs. V30M). Some Japanese patients had ocular symptoms while no Taiwanese patient had such a symptom. Dysphagia was present in half of the A97S cohort while no Japanese patients had dysphagia. The initial neuropathic symptoms in the upper limbs (excluding CTS) were more frequent in the A97S cohort than in the Japanese cohort (Théaudin et al., 2019). Furthermore, CTS appeared earlier than the length-dependent symptoms in A97S. The onset of clinical landmarks was generally earlier in the A97S cohort than the V30M cohort. In addition to genotype, these differences may imply the effect of ethnic or environmental contributions (Koike et al., 2002, 2004). The male predominance was prominent in both cohorts. This observation may be related to earlier onset in male ATTRv, reported in Portugal (Sousa et al., 1995). Female siblings of the male patients were generally asymptomatic carriers in the Japanese late-onset V30M cohort. In a mouse model, 17β-estradiol and 5α-dihydrotestosterone upregulate transthyretin synthesis (Gonçalves et al., 2008).

Various disease-modifying therapies have been developed with convincing results in these years (Castaño et al., 2012; Berk et al., 2013; Adams et al., 2018; Benson et al., 2018; Maurer et al., 2018), and the timing to initiate the therapies becomes important and depends on the natural course study for different genotypes. Furthermore, the real world data after starting the disease-modify therapies should also be compared to the natural course to study the efficacy of different disease-modifying therapies. Our study clarified several risk factors for faster progression in the early stage (FAP stage 1–2) of the A97S cohort: the early presence of dysphagia. However, the correlation was not observed in the advanced stage (FAP stage 2–3). These findings may suggest the rapid acceleration of organ damage in patients without risk factors in the advanced stage. Further study is needed to observe whether the disease-modifying therapies would slow down the organ damage speed in the advanced stage of the patients without these risk factors and whether the disease-modifying therapies would reduce the effect of the risk factors.

Our study had several limitations. First, this study was a retrospective study incorporating two cohorts from two countries, and both recall bias and selection bias existed. Some patients (37.1% in the A97S cohort) received diflunisal (Castaño et al., 2012; Berk et al., 2013), tafamidis (Maurer et al., 2018), and patisiran (Adams et al., 2018), and we right-censored the data when these patients started these therapies. Thus, the effects of disease-modifying therapies on the natural course were not studied. Finally, the swallowing functional study was only performed in ATTRA97S patients with dysphagia. It is unclear whether the functional status was different between the patients with dysphagia and those without dysphagia. In a small ATTRV30M cohort, few patients without dysphagia also had esophageal dysfunction in esophageal manometry (Bjerle et al., 1993). Further study with detailed swallowing evaluation including symptoms and examinations in patients with ATTRv with different genotypes was needed.

## Conclusion

This study demonstrates the similarity and difference in the phenotype of clinical manifestations between the two genotypes, namely, the A97S cohort and the late-onset V30M Japanese cohort. There are unique characteristics in the A97S cohort: (1) dysphagia at the early phase of ATTRv may be a risk factor of disease progression and (2) CTS potentially serves as a starting point in the continuous spectrum of amyloid neuropathy. The current approach of the phenotypical characterization with pathology examinations will serve as a foundation to analyze other genotypes for unique manifestations and underlying pathology and mechanisms. Given the development of disease-modifying therapies, these biomarkers may serve as an indicator for the early initiation of treatments.

## Data Availability Statement

The datasets generated during analysis and/or during the current study are available from the corresponding author on reasonable request.

## Ethics Statement

The studies involving human participants were reviewed and approved by (1) Ethics Committee of the National Taiwan University Hospital, Taipei, Taiwan and (2) Ethics Committee of the Nagoya University Graduate School of Medicine, Nagoya, Japan. The patients/participants provided their written informed consent to participate in this study.

## Author Contributions

H-WH, HK, and S-TH wrote the manuscript with input from all authors. All authors contributed to data acquisition and analysis.

## Conflict of Interest

The authors declare that the research was conducted in the absence of any commercial or financial relationships that could be construed as a potential conflict of interest.

## Publisher’s Note

All claims expressed in this article are solely those of the authors and do not necessarily represent those of their affiliated organizations, or those of the publisher, the editors and the reviewers. Any product that may be evaluated in this article, or claim that may be made by its manufacturer, is not guaranteed or endorsed by the publisher.
