# Supplementary material for: Unique Phenotypes With Corresponding Pathology in Late-Onset Hereditary Transthyretin Amyloidosis of A97S vs. V30M
Source: Front Aging Neurosci. 2022 Jan 26;13:786322. doi: 10.3389/fnagi.2021.786322 (PMC8826435; doi:10.3389/fnagi.2021.786322)
Supplement: Supplementary file 1 [file Table_1.docx]

| Supplementary Table 1. Laboratory data against different stages in hereditary transthyretin amyloidosis (ATTRv) | | | | | | |
| --- | --- | --- | --- | --- | --- | --- |
|  | ATTRA97S in Taiwan | | | ATTRV30M in Japan | | |
|  | Stage 1 | Stage 2 | Stage 3 | Stage 1 | Stage 2 | Stage 3 |
| Nerve conduction studies |  |  |  |  |  |  |
| Median nerve |  |  |  |  |  |  |
| Mean MCV (m/s) | 45.7 ± 0.8 | 41.5 ± 1.4 | 37.8 ± 1.3 | 44.5 ± 2.0 | 46.3 ± 2.8 | 46.1 ± 2.5 |
| Mean DL (m/s) | 5.84 ± 0.19 | 6.36 ± 0.19 | 7.16 ± 0.56 | 5.72 ± 0.38 | 4.94 ± 0.37 | 4.58 ± 0.37 |
| Mean CMAP (m/s) | 3.26 ± 0.27 | 1.75 ± 0.28 | 1.08 ± 0.28 | 3.22 ± 0.54 | 3.25 ± 0.74 | 4.04 ± 1.33 |
| Mean SCV (m/s) | 41.9 ± 1.6 | 36.0 ± 2.6 | 45.8 ± 0.8 | 47.7 ± 1.8 | 43.9 ± 2.7 | 47.9 ± 1.8 |
| Mean SNAP (uV) | 3.81 ± 0.69 | 1.95 ± 0.73 | 0.50 ± 0.23 | 2.34 ± 0.71 | 1.94 ± 0.99 | 2.68 ± 1.36 |
| Mean TLI | 0.31 ± 0.01 | 0.32 ± 0.01 | 0.32 ± 0.02 | 0.36 ± 0.02 | 0.39 ± 0.03 | 0.39 ± 0.02 |
| Tibial nerve |  |  |  |  |  |  |
| MCV (m/s) | 36.9 ±1.1 | 36.0 ± 1.4 | 33.2 ± 1.2 | 35.9 ± 1.8 | 37.6 ± 1.9 | 40.0 ± 1.7 |
| DL (m/s) | 4.93 ± 0.37 | 4.93 ± 0.22 | 5.47 ± 0.58 | 6.27 ± 0.47 | 5.30 ± 0.18 | 5.26 ± 0.82 |
| CMAP (m/s) | 2.23 ± 0.38 | 1.21 ± 0.55 | 0.12 ± 0.06 | 0.50 ± 0.21 | 0.64 ± 0.23 | 0.45 ± 0.22 |
| Sural nerve |  |  |  |  |  |  |
| SCV (m/s) | 43.4 ± 1.4 | 46.5 ± 4.0 | -^#^ | 40 ± 4.4 | 37.3 ± 2.2 | 35.7 |
| SNAP (m/s) | 1.47 ± 0.38 | 0.99 ± 0.74 | 0 | 0.57 ± 0.33 | 0.72 ± 0.44 | 0.37 ± 0.37 |
| CSF protein (mg/dL) | 62.7 ± 24.4 | 66.3 ± 31.3 | 73.2 ± 40.1 | 54.4 ± 6.9 | 49.8 ± 8.8 | 65.6 ± 9.4 |
| Data expressed as mean ± standard deviation  CSF: cerebrospinal fluid; TLI: terminal latency index; MCV: motor conduction velocity; DL: distal latency; CMAP: compound motor action potential; SCV: sensory conduction velocity; SNAP: sensory nerve action potential.  ^#^: No data | | | | | | |

| Supplementary Table 2. Laboratory data versus whether presence of dysphagia in ATTRA97S patients of stage 1. | | | | |
| --- | --- | --- | --- | --- |
|  | | Patients with dysphagia | Patients without dysphagia | *P* |
| Sonography of heart evaluation | |  |  |  |
|  | Ventricular hypertrophy | 13/17 (76.5%) | 21/28 (75%) | 0.911 |
|  | Sparkling pattern | 7/16 (43.8%) | 10/27 (37%) | 0.663 |
| Cardiac rhythm | |  |  |  |
|  | Frequent supraventricular or ventricular ectopy^a^ | 0/11 (0%) | 5/20 (25%) | 0.133 |
|  | Arrhythmia * | 10/14 (71.4%) | 15/21 (714%) | 1.000 |
| Pacemaker implantation | | 2/22 (9.1%) | 6/31 (19.4%) | 0.445 |
| Cerebrospinal fluid protein (mg/dL) | | 56.5 ± 19.3 | 67.6 ± 27.8 | 0.178 |
| Nerve conduction studies | |  |  |  |
|  | Median nerve |  |  |  |
|  | Mean MCV (m/s) | 44.8 ± 5.9 | 46.9 ± 4.3 | 0.390 |
|  | Mean DL (m/s) | 5.48 ± 1.06 | 5.99 ± 1.22 | 0.295 |
|  | Mean CMAP (m/s) | 3.01 ± 1.59 | 3.48 ± 2.15 | 0.512 |
|  | Mean SCV (m/s) | 44.3 ± 5.4 | 42.5 ± 7.1 | 0.715 |
|  | Mean SNAP (uV) | 4.33 ± 5.02 | 3.08 ± 4.92 | 0.182 |
|  | Mean Terminal latency index | 0.35 ± 0.04 | 0.30 ± 0.05 | 0.003^＃^ |
|  | Terminal latency index < 0.35 | 4/10 (40%) | 24/28 (85.7%) | 0.010^＃^ |
|  | Tibial nerve |  |  |  |
|  | MCV (m/s) | 36.1 ± 10.5 | 37.1 ± 5.2 | 0.272 |
|  | DL (m/s) | 4.12 ± 0.53 | 5.19 ± 2.64 | 0.622 |
|  | CMAP (m/s) | 3.65 ± 3.59 | 1.70 ± 1.60 | 0.131 |
|  | Sural nerve |  |  |  |
|  | SCV (m/s) | 40.9 ± 7.3 | 45.4 ± 4.8 | 0.276 |
|  | SNAP (m/s) | 2.56 ± 3.32 | 0.66 ± 2.06 | 0.001^＃^ |

^a^ Over 100 supraventricular or ventricular ectopy in one day

* Arrhythmia including atrial fibrillation, atrioventricular conduction block, or intraventricular conduction block

MCV: motor conduction velocity; DL: distal latency; CMAP: compound motor action potential; SCV: sensory conduction velocity; SNAP: sensory nerve action potential.

^＃^*P* < 0.05
